# Effect of Temperature on the Drying Kinetics of Caturra Coffee: Correlation with Hyperspectral Imaging and Sensory Quality

**DOI:** 10.3390/foods15081284

**Published:** 2026-04-08

**Authors:** Frank Fernandez-Rosillo, Nestor A. Sánchez-Goycochea, Cinthya Santa Cruz-López, Eliana Milagros Cabrejos-Barrios, Jorge Caucha-Iparraguirre, Flor Garcia-Carrión, Lenin Quiñones-Huatangari

**Affiliations:** 1 Grupo de Modelamiento y Simulación de Procesos en la Industria Alimentaria, Instituto de Investigación de Ciencia de Datos, Universidad Nacional de Jaén, Cajamarca 06800, Peru; frank_fernandez@unj.edu.pe (F.F.-R.); slopez@unj.edu.pe (C.S.C.-L.); eliana_cabrejos@unj.edu.pe (E.M.C.-B.); jorge.caucha@est.unj.edu.pe (J.C.-I.); flor.garcia@est.unj.edu.pe (F.G.-C.); 2 Centro de Apoyo Logístico al Investigador, Universidad Tecnológica del Perú, Lambayeque 14000, Peru; 3 Facultad de Ingeniería Zootecnista, Biotecnología, Agronegocios y Ciencia de Datos, Universidad Nacional Toribio Rodríguez de Mendoza, Amazonas 01001, Peru; lenin.quinones@untrm.edu.pe

**Keywords:** Caturra coffee, sensory evaluation, Biot number, Arrhenius model

## Abstract

Coffee processing requires continuous optimization to preserve sensory quality while improving process efficiency. Although hyperspectral imaging has been widely applied for food quality evaluation, its use for predicting coffee cup score during controlled drying remains limited. This study aimed to evaluate the effect of drying temperature on the drying kinetics of Caturra coffee and to develop a predictive model for cup score using hyperspectral imaging combined with Partial Least Squares Regression (PLSR). Coffee samples were dried at four constant temperatures (30, 40, 50, and 60 °C) in forced-convection ovens, and hyperspectral reflectance images (400–1000 nm) were acquired using a Specim FX10 camera. Sensory evaluation was conducted by six certified Q Arabica Graders. Drying times were 52, 34, 30, and 20 h at 30, 40, 50, and 60 °C, respectively, with corresponding cup scores of 83.21, 83.50, 83.60, and 83.26 points. Effective moisture diffusivity ranged from $10^{-13}$ to $10^{-12}$ m^2^/s, while mass transfer coefficients were on the order of $10^{-9}$ m/s, with activation energies of 28.016 and 19.272 kJ/mol. No significant differences in cup score were observed among drying temperatures (p>0.05). A PLSR-based model was developed to estimate cup score from hyperspectral data, achieving $R^{2}$ values of 0.770 and 0.855 and RMSE values of 0.515 and 0.518 for calibration and validation, respectively. Key wavelengths at 480, 600, 720, and 940 nm were identified as the most influential spectral regions associated with chemical compounds affecting sensory quality. These findings demonstrate the potential of integrating drying kinetics and hyperspectral imaging as a rapid and non-destructive approach for objective prediction of coffee sensory quality during processing.

## 1. Introduction

Coffee is one of the most economically important agricultural commodities worldwide, with global trade reaching approximately USD 138.15 billion in 2023 and projected to increase to USD 381.52 billion by 2034 [1]. In Peru, coffee is cultivated on approximately 440,000 hectares, with major varieties including Yellow Catuai, Red Caturra, Geisha, Catimor, Pache, and Bourbon, primarily produced in the Upper Jungle region under favorable agroclimatic conditions [2]. Beyond production volume, market competitiveness increasingly depends on quality differentiation.

Coffee quality is determined by sensory attributes such as aroma, flavor, aftertaste, acidity, body, and balance, which directly influence cup score and consumer acceptance [3]. These attributes result from the interaction of genetic factors, environmental conditions, agronomic practices, and post-harvest processing operations [4,5]. Among post-harvest stages, drying is particularly critical because it directly affects moisture migration, internal temperature distribution, and structural integrity of the beans.

Drying involves complex heat and mass transfer mechanisms that govern effective moisture diffusivity and mass transfer coefficients. These parameters determine moisture removal efficiency and significantly influence final bean quality. Previous studies have modeled drying kinetics to quantify moisture migration and evaluate the effects of temperature and airflow on drying performance and quality attributes [6,7]. Mathematical approaches have also been developed to estimate critical and equilibrium moisture levels under varying environmental conditions [8,9]. Although these studies provide valuable insights into drying behavior, the relationship between drying temperature and subsequent sensory quality remains insufficiently explored using rapid, non-destructive analytical techniques.

Recent studies have extensively explored drying kinetics in coffee and other agricultural products using conventional approaches based on heat and mass transfer modeling, independent of non-destructive techniques. Thin-layer and diffusion models have been widely applied to describe moisture migration and drying behavior under varying thermal conditions [10,11]. In coffee processing, drying temperature and operational conditions significantly influence effective moisture diffusivity, drying rate, and product quality [12,13]. Additionally, recent investigations have focused on optimizing drying systems and improving process efficiency through experimental and modeling approaches, including solar and hybrid drying technologies [14,15].

Despite the extensive research on drying kinetics in coffee and other agricultural products, most studies have focused on conventional modeling approaches based on heat and mass transfer principles, such as thin-layer and diffusion models, without integrating advanced sensing technologies. Recent studies have characterized moisture migration, drying rates, and effective diffusivity under different thermal conditions; however, these approaches rely on destructive or time-consuming measurements and do not enable real-time quality prediction [10,11,13].

In this context, hyperspectral imaging has emerged as a powerful analytical tool capable of capturing spectral information across a wide electromagnetic range, enabling rapid and non-destructive assessment of internal and external quality attributes [16,17]. In coffee research, hyperspectral techniques have been applied to predict chemical and aromatic compounds in roasted beans [18]. However, studies integrating hyperspectral data with sensory evaluation to predict cup score under controlled drying conditions remain limited.

Therefore, the integration of hyperspectral imaging with chemometric modeling represents a promising approach to address this gap by enabling simultaneous assessment of physicochemical transformations and sensory quality. This study hypothesizes that hyperspectral imaging combined with partial least squares regression (PLSR) enables accurate prediction of coffee cup score from spectral responses associated with physicochemical changes occurring during drying. The main scientific contribution lies in the integration of drying kinetics modeling with hyperspectral imaging to identify key spectral regions linked to sensory quality, thereby providing a framework for non-destructive monitoring of coffee processing conditions [18]. This approach addresses the limited availability of studies linking drying temperature, spectral signatures, and cup score prediction in coffee processing.

Accordingly, the aim of this study was to predict coffee cup score by linking sensory analysis with hyperspectral profiles of coffee samples dried at different temperatures. Specifically, this research sought to develop a predictive model for the sensory quality of *Coffea arabica* under varying drying conditions.

## 2. Materials and Methods

### 2.1. Data Collection

For the analysis, 12 kg of Caturra variety coffee (*Coffea arabica*) with an initial moisture content of 45% (wet basis) were selected from the 2024 harvest at the El Milagro farm, located in the district of San José de Lourdes, province of San Ignacio, department of Cajamarca, Peru (5.076986° S, 78.883632° W). The samples were divided into 12 packages of 1 kg each for subsequent experimental treatments.

### 2.2. Drying Kinetics

#### 2.2.1. Drying Condition

Drying experiments were conducted using four static-air laboratory ovens set at 30, 40, 50, and 60 °C, respectively. The selected drying temperatures represent the typical range used in controlled convective drying of coffee beans, as reported in previous studies and pilot-scale processing conditions. Temperatures around 30 °C simulate mild drying conditions similar to traditional low-temperature processing, whereas temperatures up to 60 °C are commonly applied in mechanical drying systems to accelerate moisture removal without significantly affecting sensory quality [19]. Similar temperature ranges have been widely used in drying kinetics studies of coffee to evaluate moisture diffusivity and quality attributes under hot-air drying conditions [10,20]. Therefore, this range enables the assessment of temperature effects on both mass transfer behavior and cup score under conditions representative of practical coffee processing environments. Relative humidity was monitored using a digital thermohygrometer (SH-110, Eurolab/Boeco, Germany) and maintained at approximately 60% during the drying process. Each temperature treatment was performed in triplicate.

For each replicate, 1 kg of wet coffee was uniformly distributed in a single layer on a metal mesh tray to ensure homogeneous exposure to drying conditions. The drying systems were as follows: Oven 1 (WP-25A/WP, Faithful Instrument Co., Ltd., Shanghai, China) operated at 30 °C; Oven 2 (9053, KertLab Scientific Co., Ltd., Ningbo, Zhejiang, China) at 40 °C; Oven 3 (ECOCELL, MMM Medcenter Einrichtungen GmbH, Planegg, Germany) at 50 °C; and Oven 4 (laboratory drying hood, Labtron Equipment Ltd., Camberley, UK) at 60 °C.

The initial moisture content of coffee samples was determined using the conventional gravimetric oven-drying method following AOAC reference procedures [21]. During the drying process, moisture evolution was monitored using a calibrated portable grain moisture meter (DRAMINSKI, TwistGrain Pro, Olsztyn, Poland), commonly employed by coffee producers and cooperatives for routine post-harvest quality control. Subsamples of 40 g were periodically collected from each oven, and moisture measurements were recorded every two hours [10] until a final moisture content of 12% (wet basis) was reached. This approach enabled rapid and non-destructive monitoring of moisture changes during drying without interrupting the process or altering sample conditions, while maintaining consistency with standard moisture determination practices.

Although gravimetric methods based on weight loss are commonly used for precise moisture determination, the selected method better reflects practical conditions in coffee processing and provides reliable estimates for modeling drying kinetics. Nevertheless, potential deviations associated with indirect measurements are acknowledged.

#### 2.2.2. Drying Curves

Drying curves were constructed using the recorded moisture content data as a function of drying time for each temperature treatment. The experimental data were expressed as moisture content (%, wet basis) versus time (h) to describe the drying behavior under different thermal conditions.

The curves were generated using MATLAB R2024b (MathWorks Inc., Natick, MA, USA), where data points were plotted and connected to visualize the moisture reduction trend throughout the drying process [22,23].

#### 2.2.3. Calculation of Critical and Equilibrium Moisture Content

For each of the four drying temperatures, the critical moisture content was determined from the drying rate curves derived from the experimental data [24]. The drying rate was calculated as the change in moisture content between consecutive measurements divided by the corresponding time interval.

The drying rate was plotted as a function of moisture content to identify the transition point between the constant-rate and falling-rate drying periods. The moisture content corresponding to this transition was defined as the critical moisture content.

The equilibrium moisture content was determined as the lowest moisture value reached after prolonged drying under each temperature condition. This value was considered to represent the state at which the samples exhibited negligible further moisture loss, indicating equilibrium between the product and the surrounding air.

#### 2.2.4. Mathematical Models

(A) Mass Transfer Modeling

For products with nearly spherical geometry, moisture diffusion under unsteady-state conditions can be described by Fick’s second law [25,26]. The analytical solution for a sphere is expressed as an infinite series:

(1) $$\phi \left(x\right)=\sum_{n=1}^{\infty }\frac{2\left(\sin\left(\mu_{n}\right)-\mu_{n}\cos\left(\mu_{n}\right)\right)\sin\left(\mu_{n}\frac{x}{L}\right)}{\left(\mu_{n}-\sin\left(\mu_{n}\right)\cos\left(\mu_{n}\right)\right)\mu_{n}\frac{x}{L}}$$

Coffee beans were approximated as spherical particles to simplify the analytical solution, since their geometry exhibits a nearly ellipsoidal shape with comparable characteristic dimensions. This assumption is commonly adopted in diffusion studies when geometric deviations are relatively small.

The characteristic length *L* was defined as the average measured radius of the coffee beans (m), calculated from representative samples, ϕ represents the dimensionless moisture content and $\mu_{n}$ are the positive roots associated with the Biot number.

The infinite series in Equation (1) is monotonically convergent; therefore, a satisfactory approximation can be obtained by considering only the first term of the series, denoted as μ [26]. Using this first-term approximation, the mass transfer equation for the dimensionless moisture ratio can be written as:

(2) $$\frac{M\left(x,t\right)-M_{eq}}{M_{0}-M_{eq}}=L_{1,\mu }\left(x\right)\;L_{2,\mu }\left(t\right),$$

where

(3a) $$L_{1,\mu }\left(x\right)=\frac{2\left(\sin(\mu )-\mu \cos(\mu )\right)\sin\left(\mu \frac{x}{L}\right)}{\left(\mu -\sin(\mu )\cos(\mu )\right)\mu \frac{x}{L}}$$

and

(3b) $$L_{2,\mu }\left(t\right)=\exp\left(-\frac{\mu^{2}D_{\mathrm{EFF}}}{L^{2}}t\right).$$

Here, M(x,t) is the moisture content at time *t* and position *x*; $M_{eq}$ is the equilibrium moisture content (dry basis, %); $M_{0}$ is the initial moisture content; and $D_{\mathrm{EFF}}$ is the effective moisture diffusivity coefficient (m^2^/s). Applying the natural logarithm to Equation (2) yields the linearized form:

(4) $$\ln\left(M(x,t)\right)=\ln\left(L_{1,\mu }\left(x\right)\right)-\left(\frac{\mu^{2}D_{\mathrm{EFF}}}{L^{2}}\right)t.$$

The experimental data were fitted to a linear regression model using the least squares method. The slope (*a*) and intercept (*b*) of the fitted line are given by:

(5) $$\begin{array}{c} a=-\left(\frac{\mu^{2}D_{\mathrm{EFF}}}{L^{2}}\right)\;\;\mathrm{and}\;\;b=\ln\left(\frac{2\left(\sin(\mu )-\mu \cos(\mu )\right)\sin\left(\mu \right)}{\left(\mu -\sin(\mu )\cos(\mu )\right)\mu }\right). \end{array}$$

The intercept *b* represents an approximation of $L_{1,\mu }\left(x\right)$ (Equation (3a)) under the assumption that the dimensionless radial position satisfies x/L≈1, corresponding to the external surface of the spherical particle [25].

(B) Determination of Diffusivity and Convection Coefficients and BIOT Number

Moisture transfer during drying is governed by two main transport mechanisms: internal diffusion, characterized by the effective diffusivity ($D_{\mathrm{EFF}}$), and external convective mass transfer, represented by the mass transfer coefficient ($h_{m}$). Both parameters describe mass exchange at the solid–air interface and are essential for accurately modeling drying kinetics [27,28].

An additional key parameter is the B_IOT_ number [29,30], a dimensionless quantity that relates the internal diffusion resistance within the solid to the external mass transfer resistance at its surface. This number allows evaluation of the controlling mechanism of the process and indicates whether a uniform moisture distribution inside the solid may be assumed.

Once the coefficients *a* and *b* were obtained, the parameter μ was estimated for each of the four drying temperatures by defining the function

(6) $$\begin{array}{c} g_{1}\left(\mu \right)={\hat{L}}_{1,\mu }\left(x\right)-b=0. \end{array}$$

Since the function ϕ is monotonically convergent, only the first root was sought. The nonlinear equation was solved numerically using the bisection method over the interval [0,1], performing 45 iterations and reaching a numerical error of $8.88178\cdot 10^{-16}$.

Subsequently, B_IOT_ and $D_{\mathrm{EFF}}$ were determined by solving the nonlinear equations defined in Equations (7a) and (7b), respectively [25,28]:

(7a) $$B_{\mathrm{IOT}}=1-\mu \cdot \cot(\mu ),$$

and

(7b) $$g_{2}\left(D_{\mathrm{EFF}}\right)=a+\frac{\mu^{2}}{L^{2}}D_{\mathrm{EFF}}=0,$$

where L=0.003968m corresponds to the mean radius of the coffee bean, approximated as a sphere.

Finally, the mass transfer coefficient $h_{m}$ was calculated from the relationship between the previously determined parameters:

(8) $$B_{\mathrm{IOT}}=\frac{h_{m}L}{D_{\mathrm{EFF}}}⟺h_{m}=\frac{B_{\mathrm{IOT}}\cdot D_{\mathrm{EFF}}}{L}.$$

(C) Prediction of Diffusivity and Convection Coefficients

The *D*_EFF_ and $h_{m}$ coefficients were predicted by fitting the Arrhenius model as a function of temperature [31,32,33]. The Arrhenius equation expresses a linear relationship between the natural logarithm of these mass transfer coefficients and the reciprocal of the absolute temperature of the sample, and is expressed as:

(9a) $$D_{\mathrm{EFF}}=D_{0}\;e^{-\frac{E_{a,md}}{R(T_{A}+273.15)}}⟺\ln\left(D_{\mathrm{EFF}}\right)=\ln\left(D_{0}\right)-\frac{E_{a,md}}{R(T_{A}+273.15)}$$

and

(9b) $$h_{m}=h_{0}\;e^{-\frac{E_{a,cm}}{R(T_{A}+273.15)}},⟺\ln\left(h_{m}\right)=\ln\left(h_{0}\right)-\frac{E_{a,cm}}{R(T_{A}+273.15)}$$

Here, $D_{0}$, $h_{0}$, $E_{a,md}$, and $E_{a,cm}$ are the Arrhenius constants and the activation energies for moisture diffusion and convective mass transfer, respectively; *R* is the universal gas constant (8.31446 J·mol^−1^·K^−1^), and $T_{A}$ is the drying air temperature in °C.

### 2.3. Sensory Evaluation of Coffee

The sensory evaluation of the coffee was conducted according to the cupping protocol established by the Specialty Coffee Association (SCA), with six certified Q Arabica Graders serving as panelists. The assessments were carried out under standardized environmental conditions to minimize variability and ensure the reliability of the results [34].

Cup quality (score) was determined in triplicate following a standardized cupping procedure. Caturra Coffee samples were roasted using a laboratory coffee roaster (ERTC, Industrias Metálicas de Santander—IMSA, Bucaramanga, Colombia) for 12 min under medium roast conditions, reaching a final bean temperature of approximately 200–210 °C. After roasting, samples were allowed to rest for 24 h to enable carbon dioxide degassing and stabilization of volatile compounds prior to sensory evaluation. Subsequently, 11 g of roasted coffee was weighed into each cup (55 g per treatment) and ground immediately before infusion using a laboratory grinder (Flyseago grinder, Flyseago Electric Appliance Co., Ltd., Yongkang, China). The grinding process produced a medium particle size distribution (approximately 600–850 µm), consistent with Specialty Coffee Association (SCA) recommendations for cupping analysis, ensuring uniform extraction conditions.

For beverage preparation, 1 L of filtered water was heated to 100 °C and allowed to stand for 3 min to stabilize the temperature at approximately 93 °C, in accordance with SCA recommendations. The hot water was then poured directly onto the roasted and ground coffee in Pyrex cups until full immersion was achieved. The infusion was allowed to stand for 3–5 min before breaking the crust. The tasters conducted the olfactory evaluation to identify aroma attributes, followed by gustatory assessment to evaluate flavor, acidity, body, aftertaste, balance, and overall quality. All observations were recorded using the official SCA cupping forms.

### 2.4. Hyperspectral Image Acquisition

#### 2.4.1. Acquisition of Hyperspectral Reflectance Cube of Coffee Beans

Hyperspectral images were acquired using a Specim FX10 camera (Specim, Spectral Imaging Ltd., Oulu, Finland), operating in the visible–near-infrared (Vis–NIR) range of 400–1000 nm, with a spectral resolution of approximately 5.5 nm and 112 contiguous spectral bands. The system is based on a push-broom (line-scanning) sensor, enabling simultaneous acquisition of spatial and spectral information [35].

Samples were placed on a motorized stage (40 × 21.4 cm), and illumination was provided by six halogen lamps positioned at 45° relative to the sample surface to ensure uniform lighting and minimize shadow effects. Image acquisition and system control were performed using UMO Scanner software (Specim, Spectral Imaging Ltd., Oulu, Finland), allowing synchronization between the camera and illumination system.

All images were captured under controlled laboratory conditions to ensure repeatability. Reflectance calibration was conducted prior to acquisition using white (Teflon) and dark references. The integration time and scanning speed were adjusted to optimize the signal-to-noise ratio and avoid spectral distortion.

#### 2.4.2. Reflectance Correction Using White and Dark Reference Images

Spectral data were corrected using a Teflon white reference to ensure that the measured values accurately represented the sample reflectance and minimized environmental and instrumental effects. The correction procedure followed the methodology described in [36] and was performed according to the following equation:

(10) $$Rc=\frac{S-D}{W-D},$$

where $R_{c}$ is the corrected reflectance, *S* is the raw spectral signal acquired from the sample, *D* is the dark reference signal obtained with the camera lens covered to account for sensor noise and electronic offset, and *W* is the signal acquired from a white reference panel with approximately 99.99% reflectance.

#### 2.4.3. Spectral Profile Extraction

Data segmentation was performed by selecting the region of interest (ROI). The ROI was defined using a thresholding technique to separate the coffee sample from the background, allowing extraction of the relevant spectral information [37]. For each sample, the median spectral profile within the selected ROI was calculated to obtain robust reflectance values for subsequent chemometric analysis.

#### 2.4.4. Pre-Processing of Spectral Profiles

Spectral data are commonly affected by additive and multiplicative effects, such as light scattering, instrumental variability, signal-to-noise ratio limitations, and random noise introduced during data acquisition [35]. Therefore, data pre-processing was performed to minimize these interferences and improve model robustness. Statistical analyses were conducted using the SPSS software 31.0 (IBM Corp., Armonk, NY, USA).

Before applying each pre-treatment or modeling step, reflectance values were converted to absorbance as recommended by [38], using the equation:

(11) A=−log(R),

where *A* represents absorbance and *R* corresponds to reflectance.

#### 2.4.5. Savitzky–Golay Spectral Smoothing of Hyperspectral Data

The Savitzky–Golay (SG) algorithm applies a moving window of spectral points to estimate an improved value at the central point. The window size must be optimized to balance noise reduction and signal preservation [39]. This method is considered a local polynomial smoothing filter that reduces spectral noise while preserving relevant spectral features [40].

In this study, SG filtering with a second-order polynomial and a window size of 23 points (11 points to the left and 11 to the right of the central point) was applied using MATLAB.

Following the formulation described by [41], the polynomial is defined as

(12) $$p\left(n\right)=\sum_{k=0}^{N}a_{k}n^{k},$$

where 2M+1 signal samples are considered within the moving window (with an odd number of samples), n=0 corresponds to the central point, *N* represents the polynomial order, and N≤2M to ensure a well-posed least-squares problem.

The polynomial coefficients were obtained by minimizing the mean square approximation error between the fitted polynomial and the input signal:

(13) $$E=\sum_{n=-M}^{M}{\left(\sum_{k=0}^{N}a_{k}n^{k}-x\left[n\right]\right)}^{2}.$$

This minimization allows the local signal to be smoothed while preserving its structural characteristics. The selected polynomial order (N=2) was substantially lower than the window size (2M+1=23), ensuring numerical stability while effectively reducing spectral noise.

The SG filtering reduced intensity variations caused by instrumental and scattering effects, thereby decreasing spectral dispersion and improving the uniformity of spectra acquired under identical experimental conditions [40].

### 2.5. Modeling with Partial Least Squares Regression

#### 2.5.1. Partial Least Squares Regression

PLSR modeling was performed using The Unscrambler X (version 10.4). PLSR enables the modeling of relationships between a predictor matrix (*X*) and one or more response variables (*Y*) through latent variable decomposition. Both *X* and *Y* are projected onto a new subspace defined by latent components that maximize the covariance between them. After decomposition, a regression model is constructed based on these latent variables, resulting in a predictive model [42].

The general PLSR model can be expressed as [43]:

(14) Y=βX+e,

where *Y* represents the response variable (cup score), *X* is the absorbance data matrix, β is the regression coefficient matrix, and *e* is the residual error matrix.

The optimal number of latent variables (LVs) was determined using cross-validation (5-fold). Model performance was evaluated based on the predictive coefficient of determination ($Q^{2}$), and the smallest number of components corresponding to the maximum $Q^{2}$ value was selected to avoid overfitting.

Relevant spectral bands were identified using loading plots and Variable Importance in Projection (VIP) scores. Wavelengths with high absolute loading values (loading >0.1) in the first latent components and VIP scores greater than 1.0 were considered influential variables.

Prior to modeling, spectral data were pre-processed using Savitzky–Golay smoothing and normalization. To evaluate the predictive performance and robustness of the PLSR model, the dataset was divided into calibration and independent validation subsets using a stratified sampling approach based on drying temperature. Approximately 70% of the samples were used for model calibration and 30% for external validation. Model performance was assessed using the coefficient of determination ($R^{2}$) and root mean square error (RMSE) for both datasets.

This procedure enabled evaluation of the model’s generalization capability beyond the calibration data and ensured reliable prediction of coffee cup score from spectral information.

#### 2.5.2. Spectral Absorbance Determination from Hyperspectral Reflectance Data

The conversion from reflectance to absorbance was performed based on optical principles and to enhance the predictive performance of the model. Absorbance was calculated from the corrected reflectance values obtained from the hyperspectral cube and subsequently used for model development. For comparison purposes, calibration models were also developed using the corrected reflectance values directly. Model performance was evaluated using $R^{2}$, RMSE, the selection of the optimal number of LV, and the residual predictive deviation (RPD). The RPD was calculated as the ratio between the standard deviation (SD) of the reference measurements and the RMSE of prediction:

(15) $$\mathrm{RPD}=\frac{\mathrm{SD}}{{\mathrm{RMSE}}_{\mathrm{pred}}}.$$

### 2.6. Statistical Analysis

A completely randomized design was applied to evaluate the effect of drying temperature on coffee quality parameters. Four drying temperatures (30, 40, 50, and 60 °C) were considered as treatments, each evaluated using three independent replicates. Sensory attributes and cup score values were analyzed using one-way analysis of variance (ANOVA), followed by Tukey’s multiple comparison test at a significance level of p<0.05. Prior to ANOVA, the assumptions of normality and homogeneity of variance were verified using the Shapiro–Wilk and Levene’s tests, respectively. Statistical analyses were performed using SPSS software package.

Each drying treatment was conducted in triplicate using independent experimental units to ensure reproducibility and reliability of the measured responses. This replication level is consistent with experimental designs commonly used in drying kinetics studies of agricultural products and coffee processing, where triplicate measurements provide sufficient statistical power to detect treatment effects using ANOVA while maintaining experimental feasibility [10,44]. This approach enabled reliable estimation of variability associated with moisture transport parameters and sensory quality attributes under different drying conditions.

## 3. Results

### 3.1. Modeling of Drying Kinetics

#### 3.1.1. Experimental Drying Curves

The coefficients of the linear regression functions f(t)=at+b, calculated according to Equation (5), are presented in Table 1. The magnitude of the slope (*a*) increases with temperature, indicating a faster moisture reduction rate at higher drying temperatures. The corresponding mean squared error (MSE) values range from 0.20 to 0.31, indicating that the linear approximation provides a satisfactory representation of the experimental data within the evaluated drying interval.

**Figure 1:**
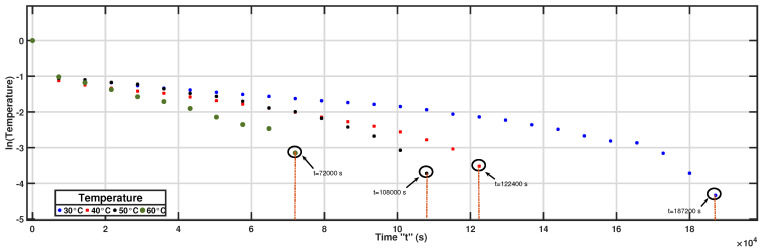
Equilibrium moisture content at 30, 40, 50, and 60 C.

Figure 1 highlights the drying times and the exact time at which the selected equilibrium moisture content on a dry basis was reached for each temperature. The recorded drying times were 52, 34, 30, and 20 h at 30, 40, 50, and 60 °C, respectively.

The curves show a rapid reduction in moisture content during the initial drying stage, followed by a gradual approach to equilibrium. The observed increase in drying time with temperature is associated with the different equilibrium moisture contents selected for each condition. Therefore, the reported times reflect the moment at which the corresponding equilibrium moisture level was reached rather than a direct comparison of intrinsic drying rates.

The reduction in drying time with increasing temperature is consistent with enhanced internal moisture diffusion driven by higher thermal gradients between the drying air and the coffee bean matrix. Elevated temperatures accelerate water migration by reducing liquid viscosity and increasing vapor pressure within the porous structure of the beans, thereby promoting faster moisture transport toward the surface.

Similar trends have been reported in convective drying studies of coffee beans, where temperature significantly influences drying rate and effective moisture diffusivity [10,20]. These findings confirm that temperature is the dominant factor governing drying kinetics under forced-convection conditions.

#### 3.1.2. Diffusivity and Convection Coefficients, and BIOT Number

Table 2 presents the estimated values of μ, $B_{\mathrm{IOT}}$, $D_{\mathrm{EFF}}$, and $h_{m}$ for the four evaluated drying temperatures (see Equations (6), (7a), (7b) and (8)).

The roots μ were determined using Equation (7a) for the corresponding $B_{\mathrm{IOT}}$ values. These roots were identified according to the vertical asymptotes of the function $\hat{B}=1-\mu \cot\left(\mu \right)-B_{\mathrm{IOT}}$ and are summarized in Table 3.

The effective moisture diffusivity values obtained in this study ($10^{-13}$–$10^{-12}$ m^2^/s) fall within the typical range reported for agricultural materials subjected to convective drying, confirming that moisture transport in coffee beans follows diffusion-controlled behavior. The increase in diffusivity with temperature reflects enhanced molecular mobility of water within the cellular structure and reduced resistance to internal mass transfer.

Similar diffusivity ranges have been reported for coffee drying under controlled hot-air conditions, supporting the validity of the diffusion-based modeling approach applied in this work [10,45].

The estimated convective mass transfer coefficients were on the order of $10^{-9}$ m/s, indicating efficient external moisture removal during forced-air drying. These values are consistent with those reported for thin-layer drying of coffee and other biological materials, where external resistance to mass transfer decreases as drying temperature increases.

This behavior suggests that internal diffusion mechanisms played a more dominant role than surface evaporation in controlling the drying process under the evaluated conditions.

#### 3.1.3. Prediction of Diffusivity and Convection Coefficients Using Arrhenius

Table 4 shows the estimated Arrhenius model parameters along with their correlation coefficients. The activation energy values obtained in this study (19.272–28.016 kJ/mol) fall within the typical range reported for moisture diffusion in food materials subjected to convective drying. These values indicate a moderate sensitivity of moisture transport to temperature variations and confirm that the drying process is primarily governed by diffusion mechanisms rather than structural limitations. Comparable activation energy values have been reported for coffee beans and other agricultural products under similar drying conditions [45,46].

**Figure 2:**
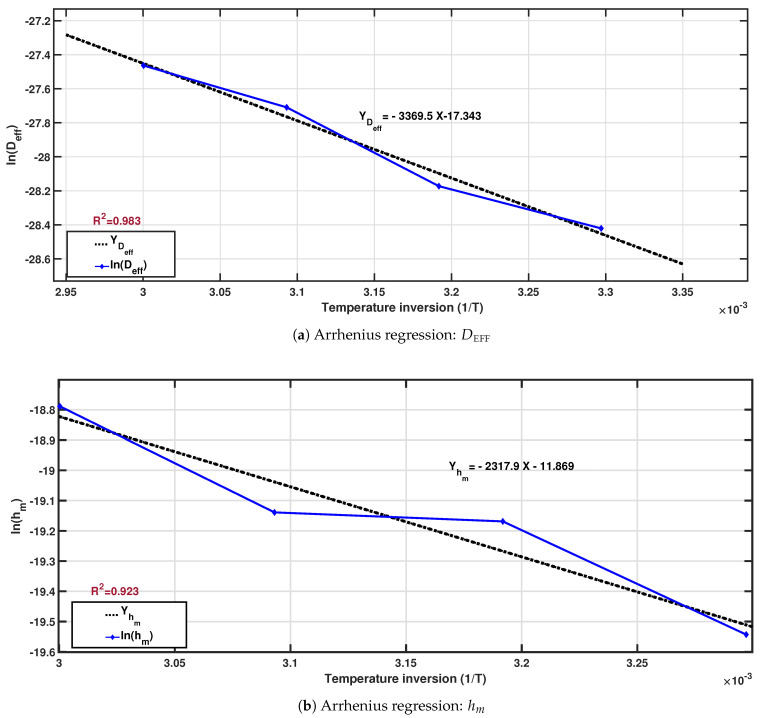
Linear regressions according to the Arrhenius model for the effective diffusivity ($D_{\mathrm{EFF}}$) and mass transfer coefficient ($h_{m}$). (**a**) Regression for $D_{\mathrm{EFF}}$. (**b**) Regression for $h_{m}$.

The regression graphs corresponding to the Arrhenius model for both the effective diffusion coefficient ($D_{\mathrm{EFF}}$) and the mass transfer coefficient ($h_{m}$) are presented in Figure 2.

The importance of this relationship lies in providing an estimate of the activation energy required for moisture transfer during drying. Higher activation energy indicates that water molecules are more strongly bound within the product structure, affecting drying time [28]. Based on the equations in (9), the predicted values of $D_{\mathrm{EFF}}$ and $h_{m}$ at different temperatures are summarized in Table 5.

### 3.2. Sensory Analysis

The results of the sensory analysis (cup score) of Caturra coffee were obtained by six certified tasters, who assigned values between 0 and 10 to each attribute presented in Table 6. The cup scores ranged from 80 to 88 points among the treatments. The sample dried at 50 °C achieved the highest score, 83.60 points.

The ANOVA was conducted to evaluate the effect of drying temperature on cup score. The results showed no statistically significant differences among treatments (F=0.049, p=0.985), indicating that drying temperature within the evaluated range (30–60 °C) did not significantly affect the sensory quality of coffee.

### 3.3. Partial Least Squares Regression Model Prediction

**Figure 3:**
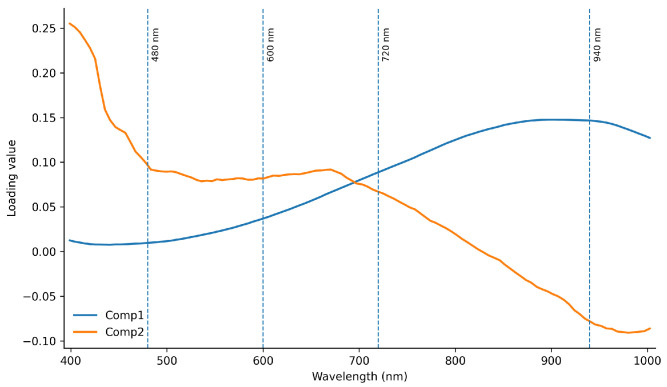
Loading plots of the first two components (Comp1 and Comp2) derived from the spectral dataset. The most influential wavelengths are observed around 480, 600, 720, and 940 nm, indicating their contribution to spectral variability associated with coffee cup score. These regions are associated with key chemical constituents, including phenolic compounds, Maillard reaction products, and moisture-related absorptions, which play a critical role in sensory quality.

The loading plots of the first two components (Comp1 and Comp2) derived from the spectral dataset (Figure 3) identify the key wavelengths contributing to spectral variability associated with coffee cup score. The most influential regions are observed around 480, 600, 720, and 940 nm, as indicated by peaks in the loading profiles. These wavelengths correspond to spectral regions with higher contributions to the model, suggesting their relevance in capturing chemical variations related to sensory attributes.

**Figure 4:**
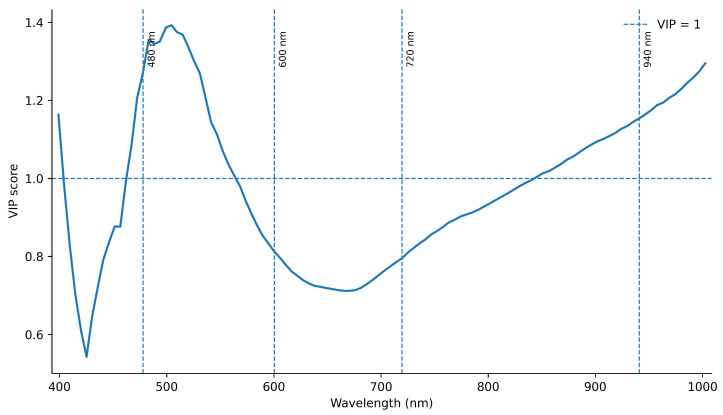
Variable importance in projection (VIP) scores derived from the spectral dataset. The horizontal dashed line (VIP = 1) indicates the threshold for significant variables. Wavelengths around 480, 600, 720, and 940 nm exceed this threshold, highlighting their relevance in predicting coffee cup score.

The Variable importance in projection (VIP) plot (Figure 4) illustrates the relative contribution of each wavelength to the PLSR model. Wavelengths with VIP values greater than 1 were considered significant contributors to the prediction of coffee cup score. The most relevant spectral regions are observed around 480, 600, 720, and 940 nm, confirming their importance in explaining sensory variability.

**Figure 5:**
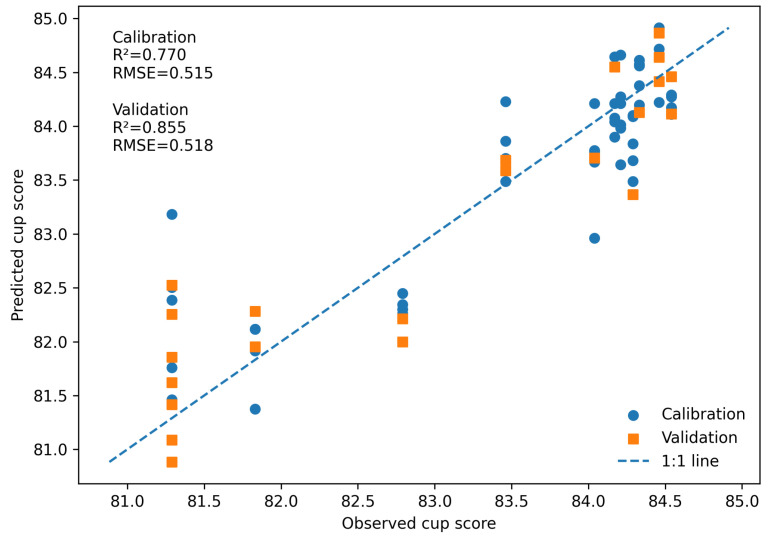
Predicted versus observed cup score values obtained from the PLSR model using five latent variables. Circles represent calibration samples, and squares represent validation samples. The dashed line indicates the 1:1 relationship between predicted and observed values. The model shows good predictive performance, with $R^{2}$ values of 0.770 and 0.855 and RMSE values of 0.515 and 0.518 for calibration and validation, respectively.

Figure 5 shows the actual modeling compared to the PLSR prediction of the cup quality profile with the spectral profiles, demonstrating the accuracy of the PLSR model in predicting the cup profile. Blue points correspond to the training set, while red points represent the validation set.

The proximity of the data points to the 45° line indicates that the model accurately captures the relationship between spectral profiles and sensory scores. The performance metrics shown in the figure further support this interpretation. The RMSE values (0.515 for training and 0.518 for validation) are relatively low, indicating good predictive performance, while the $R^{2}$ values (0.770 for training and 0.855 for validation) demonstrate that the model explains a substantial proportion of the variance in cup scores.

Overall, these results confirm that the PLSR model provides reliable predictions of coffee quality, with acceptable generalization to unseen samples.

The optimal PLSR model was constructed using five latent variables, as determined by cross-validation. However, only the first two components were considered for graphical representation (loading and score plots), as they capture the main variance structure and facilitate interpretation of spectral contributions. The remaining components contribute to model refinement but have a limited impact on visualization and interpretation.

Equation (16) shows the prediction model for the response variable (cup score) for the sensory score.

(16) Y=83.14+0.38·Comp1−0.21·Comp2

where

$$\begin{array}{cc} Comp1 & =+0.15\cdot Abs480+0.09\cdot Abs600-0.12\cdot Abs720+0.07\cdot Abs940,\\ Comp2 & =-0.08\cdot Abs480+0.13\cdot Abs600+0.05\cdot Abs720-0.04\cdot Abs940, \end{array}$$

and Abs480,Abs600,Abs720, and Abs940 are the absorbance values for a specific coffee sample at wavelengths of 480, 600, 720 and 940 nm, regardless of the drying temperature (range between 30 and 60 °C).

## 4. Discussion

The results confirm that drying temperature strongly influences moisture removal kinetics in coffee beans. When the temperature increased from 30 to 60 °C, drying time decreased from 54 h to 22 h, demonstrating the dependence of mass transfer processes on thermal gradients. This behavior is associated with the increase in vapor pressure differences between the internal moisture of the bean and the surrounding air, which accelerates moisture migration and evaporation. Similar trends have been widely reported in drying studies of agricultural products, where higher temperatures enhance moisture diffusivity within porous food matrices [47,48,49].

These findings are consistent with recent studies on drying kinetics of coffee and agricultural products, where temperature has been identified as a critical factor influencing moisture diffusivity and drying rate. For example, ref. [10] reported that increasing drying temperature significantly enhances moisture removal efficiency and reduces drying time in coffee processing. Similarly, ref. [13] that higher temperatures promote greater effective diffusivity due to inocreased molecular mobility of water within the prduct matrix. These results confirm that the observed behavior in this study follows well-established diffusion-driven drying mechanisms.

The estimation of Biot parameters and effective diffusivity coefficients provides insight into the heat and mass transfer mechanisms during coffee drying. The predicted effective moisture diffusivity ($D_{\mathrm{EFF}}$) ranged between $10^{-13}$ and $10^{-12},m^{2}/s$, which falls within the typical range reported for convective drying of biological materials. These values indicate that internal diffusion is the dominant mechanism governing moisture transport within the coffee bean matrix. Likewise, the estimated convective mass transfer coefficients ($h_{m}$), on the order of $10^{-9}m/s$, reflect the contribution of external mass transfer to the drying process. Together, these parameters offer a quantitative basis for modeling and optimizing drying systems.

Although moisture content was determined using a portable moisture meter, the estimated diffusivity values fall within the typical range reported for convective drying of agricultural materials [50,51], generally between $10^{-11}$ and $10^{-9}$ m^2^/s. This agreement suggests that the measurement approach does not compromise the physical interpretation of moisture transport phenomena. Similar ranges have been consistently reported in drying studies of food materials, where effective moisture diffusivity typically varies from $10^{-12}$ to $10^{-9}$ m^2^/s depending on processing conditions and product structure [52,53]. Moreover, the dependence of diffusivity on temperature and moisture content has been widely confirmed, supporting the robustness of Fickian diffusion-based models as a reliable framework for describing moisture migration during convective drying [54].

Activation energy is also a key indicator of moisture diffusion behavior. The estimated activation energies of 28.016 kJ/mol for $D_{\mathrm{EFF}}$ and 19.272 kJ/mol for $h_{m}$ suggest moderate sensitivity of moisture transport to temperature changes. These values are consistent with those reported for several agricultural products and represent the energy required for water molecules to migrate through the product structure during drying [55,56]. From a practical standpoint, this indicates that moderate increases in temperature can significantly improve drying efficiency without excessive energy requirements.

Equilibrium moisture content showed a decreasing trend as temperature increased, indicating a reduction in the binding strength between water molecules and the coffee matrix. This phenomenon is associated with the weakening of intermolecular forces within the porous structure of the beans. In addition, critical moisture content values ranged from 22.97 to 23.83%, corresponding to the transition from the constant-rate period to the falling-rate drying stage, where internal diffusion becomes the dominant transport mechanism. Similar drying behavior has been reported in studies on coffee and other agricultural commodities [57,58,59].

The absence of significant differences in cup score (p>0.05) indicates that drying temperature within the evaluated range does not play a determining role in sensory quality, suggesting that other factors, such as post-harvest processing or intrinsic bean characteristics, may exert a greater influence. All treatments achieved values above 83 points, maintaining the classification of specialty coffee. This suggests that moderate variations in drying temperature do not substantially alter the chemical compounds responsible for flavor and aroma development. Previous studies have similarly reported that drying conditions within moderate temperature ranges have limited influence on the final sensory profile of coffee [60,61,62]. Nevertheless, small variations observed in attributes such as aroma, acidity, and aftertaste highlight the complex interactions between processing conditions, genetic characteristics, and environmental factors influencing coffee quality [63,64]. Largo-Avila et al. [12] reported that controlled hot-air drying preserves key sensory attributes, suggesting that quality degradation is more closely associated with extreme processing conditions than with moderate thermal variations. These findings support the conclusion that drying temperature alone is not a determining factor for sensory quality within the evaluated range.

The application of hyperspectral imaging represents a promising approach for analyzing coffee quality attributes. By capturing reflectance spectra within the 400–1000 nm range, hyperspectral techniques allow the identification of chemical and structural variations related to coffee composition. The PLSR model established predictive relationships between spectral absorbance and cup quality indicators, supporting previous studies where spectral data has been successfully applied for food quality prediction [65]. Similar approaches have been used in coffee research to identify spectral regions associated with important chemical compounds and quality markers [66,67].

The VIP analysis reinforces the relevance of the selected spectral regions, showing that wavelengths around 480 and 940 nm, together with adjacent regions, contribute most strongly to the predictive model. These bands are chemically meaningful, as they are associated with phenolic compounds, process-related chromophores, and water-related absorptions commonly identified using visible and near-infrared spectroscopy in food systems [68,69]. In particular, hyperspectral imaging combined with chemometric approaches such as PLSR has demonstrated strong capability for predicting chemical and sensory attributes in coffee and other food matrices, highlighting the importance of wavelength selection for model performance [18,70]. The agreement between the VIP profile and the loading interpretation supports the robustness and interpretability of the hyperspectral model, indicating that variable selection reflects meaningful chemical information rather than statistical artifacts.

The loading plots (Figure 3) enabled the identification of key wavelengths contributing to the prediction of coffee cup score, particularly around 480, 600, 720, and 940 nm. From a physicochemical perspective, these wavelengths are associated with specific compounds influencing coffee quality. The region around 480 nm is linked to chromophoric compounds, such as chlorogenic acids and other phenolic constituents, which affect bitterness and astringency [71]. The bands in the 600–720 nm range are associated with Maillard reaction products and melanoidins formed during processing, contributing to color and flavor development and [72,73]. In contrast, the absorption feature around 940 nm corresponds to O–H stretching overtones, reflecting moisture content and water-related interactions within the coffee matrix, as commonly observed in near-infrared spectroscopy [74,75]. The consistency of these spectral features with known chemical attributes supports the robustness of the spectral model and highlights the potential of hyperspectral imaging for predicting coffee sensory quality.

The two latent components extracted by the PLSR model explained a significant proportion of the variance in the dataset, demonstrating the potential of hyperspectral information for predicting coffee cup quality. Although the predictive performance ($R^{2}\approx 0.68$) indicates moderate accuracy, the model still shows practical value as a rapid and non-destructive evaluation tool [76,77].

The results obtained in this study are consistent with previous research demonstrating the potential of spectral techniques for predicting sensory quality in coffee. Hyperspectral and near-infrared spectroscopy, combined with chemometric models such as PLSR, have been successfully applied to estimate aroma, flavor, and overall cup score, highlighting their effectiveness as rapid and non-destructive tools [18,78]. These approaches enable the identification of relevant spectral regions associated with chemical compounds that influence sensory perception, thereby improving model interpretability and predictive performance [70,79]. Compared with conventional sensory evaluation, which is time-consuming and subject to variability, spectral methods provide objective and reproducible measurements, making them suitable for industrial applications in coffee quality control. The agreement between the present results and previous studies reinforces the reliability of hyperspectral imaging as an effective tool for predicting coffee cup score.

From a practical perspective, integrating drying kinetics, hyperspectral analysis, and sensory evaluation provides a comprehensive framework for optimizing post-harvest coffee processing. The ability to estimate critical moisture levels (approximately 23%) and equilibrium moisture levels (around 12%) through spectral information could enable real-time monitoring of drying operations. Moreover, the combined results suggest that operating within a temperature range of 40–50 °C may represent an optimal balance between drying efficiency and quality preservation, reducing drying time while maintaining specialty coffee cup scores above 83 points.

Unlike conventional drying studies that rely primarily on physicochemical measurements, this study integrates hyperspectral imaging with drying kinetics and sensory analysis, providing a comprehensive and non-destructive framework for quality prediction. Previous research has mainly focused on optimizing drying parameters or modeling moisture transfer independently [11,14]; in contrast, the present approach establishes a direct link between spectral data and sensory attributes. This integration represents a relevant advancement, as it enables rapid prediction of coffee quality and supports real-time monitoring and decision-making in industrial applications.

## 5. Conclusions

The combined modeling of drying time, equilibrium and critical moisture contents, together with the estimation of $D_{\mathrm{eff}}$ and $h_{m}$ coefficients, provided a consistent framework for describing moisture dynamics and thermodynamic effects during coffee drying. Drying temperature significantly affected drying kinetics, reducing drying time from 52 h at 30 °C to 20 h at 60 °C. However, no statistically significant differences in cup score were observed within the evaluated temperature range (30–60 °C), indicating that temperature alone does not determine coffee sensory quality.

The estimated transport coefficients were within the typical ranges reported for agricultural materials ($D_{\mathrm{eff}}$ between $10^{-13}$ and $10^{-12}$ m^2^/s and $h_{m}$ on the order of $10^{-9}$ m/s), supporting the suitability of the diffusion-based approach for describing moisture migration in Caturra Coffee. In addition, the integration of hyperspectral imaging with PLSR enabled the development of a predictive model capable of estimating cup score from spectral data with satisfactory accuracy.

Nevertheless, the model was developed using a limited dataset under controlled experimental conditions, which may restrict its applicability to other processing environments. Moreover, the use of a single coffee variety limits the generalization of the results to different cultivars, processing methods, or environmental conditions. Future research should evaluate the robustness of the proposed approach using larger and more diverse datasets, including independent samples from different post-harvest scenarios.

Overall, the integration of drying kinetics, hyperspectral imaging, and chemometric modeling represents a promising non-destructive strategy for supporting quality monitoring during coffee drying, although further validation is required prior to large-scale industrial implementation. 

## Figures and Tables

**Table 1:** Linear regression coefficients of the drying curves at different temperatures.

Temperature (°C)	$a\times 10^{-5}$	*b*	MSE
30	−1.46982169	−0.59745164	0.30508460
40	−2.01117947	−0.67151457	0.23127388
50	−2.48775171	−0.44401679	0.26330223
60	−3.39803574	−0.49186182	0.21446990

**Table 2:** Estimated parameters μ, $B_{\mathrm{IOT}}$, $D_{\mathrm{EFF}}$, and $h_{m}$ at different drying temperatures.

Temperature (°C)	μ	$D_{\mathrm{EFF}}\times 10^{-13}$ (m^2^/s)	Biot	$h_{m}\times 10^{-9}$ (m/s)
30	2.256442	4.538592	2.845864	3.255095
40	2.333156	5.817569	3.228052	4.732716
50	2.058683	9.245937	2.092496	4.875778
60	2.127121	11.821655	2.322733	6.919997

**Table 3:** Roots $\mu_{n}$ associated with each $B_{\mathrm{IOT}}$ value.

Biot	$\mu_{1}$	$\mu_{2}$	$\mu_{3}$	$\mu_{4}$	$\mu_{5}$	$\mu_{6}$
2.8459	2.2564	5.0621	8.0786	11.1595	14.2659	17.3845
3.2281	2.3332	5.1227	8.1217	11.1921	14.2918	17.4061
2.0925	2.0587	4.9304	7.9899	11.0937	14.2139	17.3417
2.3227	2.1271	4.9724	8.0175	11.1140	14.2299	17.3548

**Table 4:** Estimated Arrhenius model parameters for effective diffusivity ($D_{\mathrm{EFF}}$) and mass transfer coefficient ($h_{m}$).

Coefficient	Regression (y=ax+b)	Factor $D_{0}$ (m^2^/s) or $h_{0}$ (m/s)	Activation Energy $E_{a}$ (kJ/mol)	$R^{2}$
Equation (9a) $D_{\mathrm{EFF}}$	a=−3369.5,b=−17.343	$2.939\cdot 10^{-8}$	28.016	0.983
Equation (9b) $h_{m}$	a=−2317.9,b=−11.869	$7.006\cdot 10^{-8}$	19.272	0.923

**Table 5:** Predicted values for $D_{\mathrm{EFF}}$ and $h_{m}$ at different temperatures.

Temperature (°C)	$D_{\mathrm{EFF}}$ (m^2^/s)	$h_{m}$ (m/s)
25	$3.652\cdot 10^{-13}$	$2.957\cdot 10^{-9}$
35	$5.268\cdot 10^{-13}$	$3.804\cdot 10^{-9}$
45	$7.426\cdot 10^{-13}$	$4.818\cdot 10^{-9}$
55	$1.025\cdot 10^{-12}$	$6.014\cdot 10^{-9}$
65	$1.389\cdot 10^{-12}$	$7.410\cdot 10^{-9}$
70	$1.605\cdot 10^{-12}$	$8.188\cdot 10^{-9}$

**Table 6:** Sensory attributes and cup scores.

Attributes	Temperature			
	30 °C	40 °C	50 °C	60 °C
Fragrance	7.61±0.32 *^a^*	7.76±0.23 *^a^*	7.67±0.12 *^a^*	7.68±0.23 *^a^*
Flavor	7.57±0.28 *^a^*	7.58±0.22 *^a^*	7.68±0.15 *^a^*	7.54±0.23 *^a^*
Aftertaste	7.54±0.27 *^a^*	7.54±0.18 *^a^*	7.58±0.19 *^a^*	7.53±0.27 *^a^*
Acidity	7.57±0.17 *^a^*	7.71±0.22 *^a^*	7.68±0.06 *^a^*	7.69±0.23 *^a^*
Body	7.63±0.18 *^a^*	7.64±0.20 *^a^*	7.64±0.10 *^a^*	7.65±0.25 *^a^*
Uniformity	9.89±0.19 *^a^*	10.0±0.00 *^a^*	10.0±0.00 *^a^*	10.0±0.00 *^a^*
Balance	7.69±0.34 *^a^*	7.61±0.21 *^a^*	7.61±0.13 *^a^*	7.61±0.29 *^a^*
Clean cup	10.0±0.00 *^a^*	10.0±0.00 *^a^*	10.0±0.00 *^a^*	10.0±0.00 *^a^*
Sweetness	10.0±0.00 *^a^*	10.0±0.00 *^a^*	10.0±0.00 ^a^	10.0±0.00 *^a^*
Taster score	7.71±0.29 *^a^*	7.76±0.23 *^a^*	7.67±0.13 *^a^*	7.68±0.23 *^a^*
Cup score	83.2±1.66 *^a^*	83.5±1.45 *^a^*	83.6±0.88 *^a^*	83.26±1.71 *^a^*

Note 1: ±: standard deviation of 18 data points (three replicates from each of the six tasters). Note 2: Different letters indicate significant differences (p<0.05).

## Data Availability

The original contributions presented in this study are included in the article. Further inquiries can be directed to the corresponding author.
